# Supplementary material for: Synthetic algal-bacteria consortia for space-efficient microalgal growth in a simple hydrogel system
Source: J Appl Phycol. Author manuscript; Available in PMC 2024 Dec 10. (PMC7617206; doi:10.1007/s10811-021-02528-7)
Supplement: Supplementary Materials [file EMS188108-supplement-Supplementary_Materials.docx]

**Supplementary information**

**Synthetic algal-bacteria consortia for space-efficient microalgal growth in a simple hydrogel system**

Noah Martin^1^*, Tatum Bernat^1^*, Julie Dinasquet^1^, Andrea Stoftko^1^, April Damon^1^, Dimitri D. Deheyn^1^, Farooq Azam^1^, Jennifer E. Smith^1^, Matthew P. Davey^2,3^ Alison G. Smith^2^, Silvia Vignolini^4^, & Daniel Wangpraseurt^1,4,5#^

^1^Marine Biology Research Division, Scripps Institution of Oceanography, University of California San Diego, La Jolla, California 92093-0205, USA

^2^Department of Plant Sciences, University of Cambridge, Cambridge CB2 3EA, UK

^3^Scottish Association for Marine Science, Oban, PA37 1QA, UK

^4^Department of Chemistry, University of Cambridge, Cambridge CB2 1TN, UK

^5^Department of Nanoengineering, University of California San Diego, La Jolla, California 92093-0205, USA

*shared first author

#correspondance: [dwangpraseurt@eng.ucsd.edu](mailto:dwangpraseurt@eng.ucsd.edu) (D. Wangpraseurt)

| **Supplementary Table 1** | |
| --- | --- |
| Bacterial isolates tested from our culture collection. Closest relative assignment based on 16S rRNA gene sequencing. All bacteria were isolated off Scripps Pier. | |
|  |  |
| **Bacterial strain** | **Closest relative** |
| SIO_BE5 | AlphaProteobacteria; Rhodobacterales; Rhodobacteraceae; *Phaeobacter* sp. |
| SIO_La7 | AlphaProteobacteria; Hyphomonodales; Hyphomonodaceae; *Henriciella* sp |
| SIO_La6 | AlphaProteobacteria; Sphingomonadales; Erythrobacteraceae; *Erythrobacter* sp. |
| SIO_La5 | AlphaProteobacteria; Rhodobacterales; Rhodobacteraceae; *Phaeobacter* sp. |
| SIO_La4 | AlphaProteobacteria; Rhodobacterales; Rhodobacteraceae; *Phaeobacter* sp. |
| SIO_La1 | AlphaProteobacteria; Sphingomonadales; Erythrobacteraceae; *Erythrobacter* sp. |
| B6P1 | Gammaproteobacteria; Alteromonadales; Alteromonadaceae; *Alteromonas macleodii* |
| AltSIO | Gammaproteobacteria; Alteromonadales; Alteromonadaceae; *Alteromonas macleodii* |
| P1RIIB2 | Bacteroidetes; Flavobacteriales; Flavobacteriaceae; *Cellulophaga* sp. |
| BBFL7 | Bacteroidetes; Flavobacteriales; Flavobacteriaceae; *Flavobacterium* sp. |
| DMS2 | Gammaproteobacteria; Oceanospirillales; Oceanospirillaceae; *Marinomonas* sp. |
| A1.2 | Bacteroidetes; Flavobacteriales; Flavobacteriaceae; *Polaribacter* sp. |
| MMK1 | Alphaproteobacteria; Rhizobiales; Methylobacteriaceae; *Methylobacterium* sp. |
| TW7 | Gammaproteobacteria; Alteromonadales; Pseudoalteromonadaceae; *Pseudoalteromonas* sp. |

**Supplementary Table 2**

Statistical analyses performed on the effect of monoculture vs. co-culture on *Marinichlorella kaistiae* KAS603 hydrogels (unpaired t-tests, α=0.05, *= significant difference).

| **Parameter** | **t** | **df** | **Sig. (2-tailed)** | **Mean difference** |
| --- | --- | --- | --- | --- |
| *cells day 1* | 1.101 | 6 | 0.313 | 405000 |
| *cells day 2* | 2.836 | 7 | 0.025* | -3689091 |
| *cells day 3* | 5.176 | 7 | 0.001* | -16659091 |
| *O_2_ flux, dark* | 22.151 | 3 | 0.0002* | 0.0371 |
| *O_2_ flux, light* | 6.117 | 3 | 0.008* | -0.054 |
| *F_v_/F_m_ day 2* | 2.578 | 4 | 0.061 | -0.063 |
| *F_v_/F_m_ day 3* | 3.216 | 4 | 0.032* | -0.142 |
| *F_v_/F_m_ day 7* | 5.289 | 4 | 0.0006* | -0.068 |


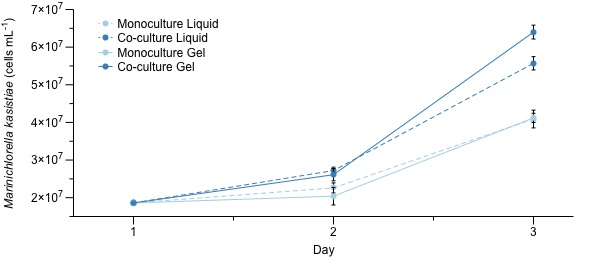


**Supplementary Fig. 1** Comparison of cell growth of *Marinichlorella kaistia*e KAS603 in co-culture with Erythrobacter strain SIO_LA6 (dark blue) and in mono-culture (light blue) when cultivated in liquid medium vs hydrogel. Data are means ± SE (*n* = 2-3 hydrogels/liquid culture flasks)


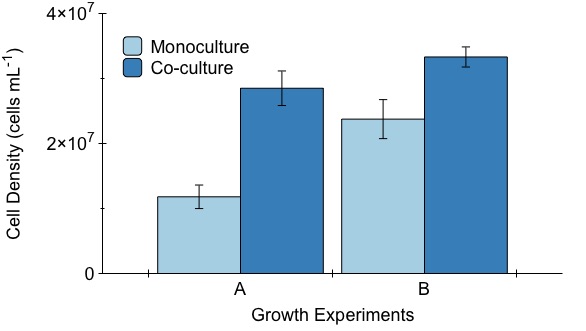


**Supplementary Fig. 2** Effect of algal-bacterial hydrogel co-culture on microalgal cell density growth after 72 hours. Microalgal starting cell density was 5.42 x 10^6^ (± 4.2 x 10^5^) cells/mL for growth experiment A and 2.21 x 10^7^ (± 4.9 x 10^5^) cells/mL for growth experiment B. Data are means ± SE (*n* = 2 hydrogels for monoculture B, *n* = 5 for all other groups).


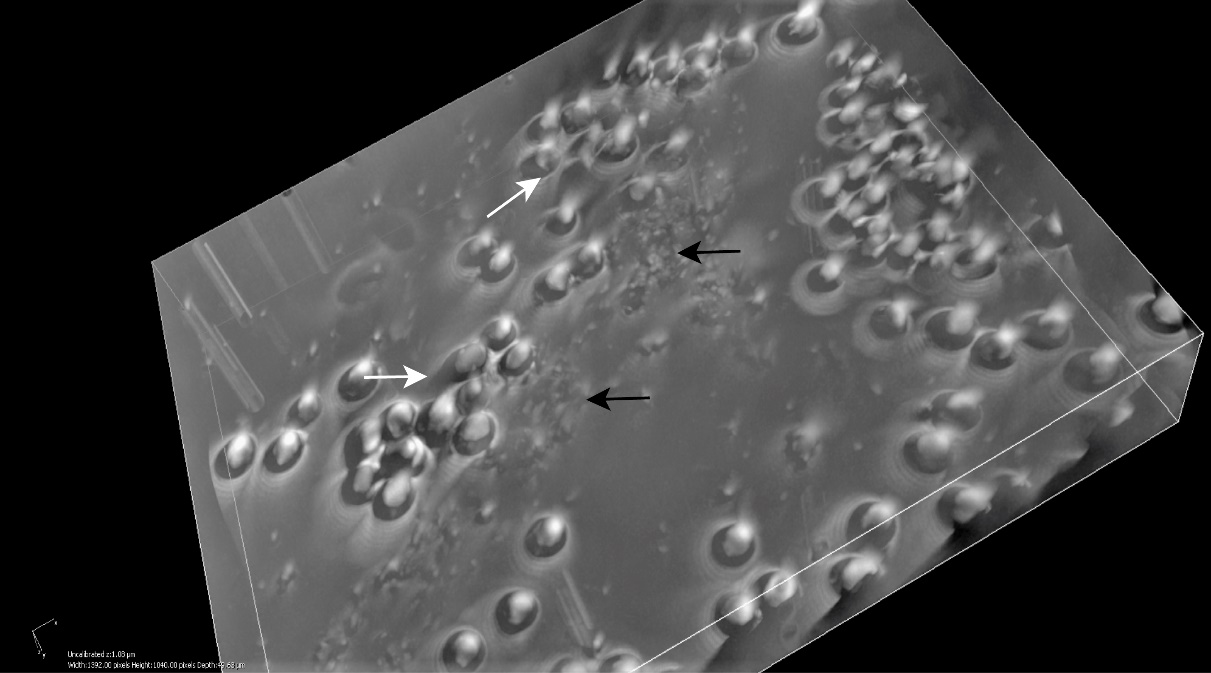


**Supplementary Fig. 3** 3D composite image of co-culture in hydrogel. White arrows show the *M. kaistiae* aggregates above the bacterial SIO_La6 aggregates (black arrows). (Brightfield magnification 200x)
